# The Unique Contribution of Past Bullying Experiences to the Presence of Psychosis-Like Experiences in University Students

**DOI:** 10.3389/fpsyt.2022.839630

**Published:** 2022-04-28

**Authors:** Jie Zhao, Xiao-Hua Lu, Yuan Liu, Nan Wang, Dong-Yang Chen, Iun-An Lin, Xiao-Hong Li, Fu-Chun Zhou, Chuan-Yue Wang

**Affiliations:** ^1^The National Clinical Research Center for Mental Disorders and Beijing Key Laboratory of Mental Disorders Beijing Anding Hospital and the Advanced Innovation Center for Human Brain Protection, School of Mental Health, Capital Medical University, Beijing, China; ^2^Psychological Consulting Center, Beijing Jiaotong University, Beijing, China; ^3^Nursing Department, National University Polyclinic, Singapore, Singapore; ^4^Department of General Psychiatry, Central Region, Institute of Mental Health, Singapore, Singapore

**Keywords:** bullying, childhood trauma and adversity, college students, psychosis-like experiences, psychometric properties

## Abstract

**Objectives:**

Both bullying and psychosis-like experiences (PLEs) have gained much attention in recent years, but their interactions are not fully unraveled. The aim of the current study was to validate the Chinese version of Bullying Scale for Adults (C-BSA), and to investigate whether past bullying experiences independently predict the presence of PLEs in university students.

**Methods:**

The validity and reliability of the C-BSA were determined in two independent samples. A battery of psychological inventories was also administered to assess the presence of PLEs, maltreatment history in the family, and current depression and anxiety, including the 15-item positive subscale of the community assessment of psychic experiences (CAPE-p15), the Chinese version of the Childhood Trauma Questionnaire (CTQ), Self-Rating Depression Scale (SDS), and Self-Rating Anxiety Scale (SAS).

**Results:**

In the construction sample (*N* = 629), a Cronbach's α of 0.921 indicated a good internal consistency of C-BSA. The exploratory factor analysis (EFA) yielded a four-factor model and a three-factor model, and both were verified by using the confirmatory factorial analysis (CFA) in the validation sample (*N* = 629). The total scores of C-BSA were significantly correlated with that of CTQ, CAPE-p15, SDS, and SAS. Multivariate logistic regression revealed that bullying was associated with 2.0 or 3.7 times of risk for the presence of PLEs (numbers of bullying types < = 3 or > 3, respectively) after controlling for CTQ, SDS, and SAS scores.

**Conclusions:**

C-BSA has shown good psychometric properties in college students. The contribution of past bullying experiences to the present PLEs seems to be independent of other childhood trauma, current depression, and anxiety.

## Introduction

It had been believed that psychosis was a categorical concept and a clear boundary existed between psychotic and Non-psychotic status. However, the conception of continua of human behaviors has received much attention in past years. The crucial role of both functional and dysfunctional behaviors on the continua has been discussed both in clinical contexts and in neuroscientific and neuroevolutionary contexts (1, 2). Recent years have seen an increasing trend to consider psychosis as a continua (3), ranging from psychosis-like experiences (PLE) (or psychotic-like experiences), clinical high risk for psychosis (CHR), to full-blown psychosis.

The term PLEs is widely used to describe transitory hallucinatory and (or) delusional experiences that are below the threshold of clinical psychosis. According to a systematic review by Lee et al. (4), PLEs have been defined by three different approaches, including (i) preset criteria, (ii) assessment tools with predetermined threshold (e.g., Comprehensive assessment of at-risk mental states, CAARMS), and (iii) assessment tools quantitatively without predefined criteria (e.g., Community Assessment of Psychic Experiences, CAPE) (4). PLEs are frequently reported in the community with a prevalence rate of 5–8%. PLEs have also been linked to an increased risk of developing psychotic disorders as well as suicidal behavior in the future (5–7).

For the past few years, the role of trauma on the psychosis continuum has gained increasing attention. Meta-analyses have revealed that childhood adversities are related to an increased risk for developing psychosis and persistence of psychotic-like experiences (8, 9). Dose-responses between trauma and psychotic symptoms or Non-clinical psychotic-like experiences were found in different research, and this makes the role of trauma hard to be ignored (10–15). Psychotic patients with childhood adversities tend to have declined psychosocial functioning, more severe clinical symptoms, longer hospital stays, lower self-rated life quality scores, as well as slower symptomatic remission rates over time (16–20).

In consideration of the variety of trauma types, various measurements are taken to capture the different dimensions of trauma. Many investigators use either some modules from structured interviews (e.g., The Composite International Diagnostic Interview, CIDI) or single scale (e.g., The Short version of the Childhood Trauma Questionnaire, CTQ) to assess trauma, however, only specific aspects of trauma (21, 22) are covered. For instance, most of the five dimensions of CTQ only inquire about the abuses and neglects which happen within the family context. However, school and workplace bullying events are also traumatic and this needs to be assessed independently.

Bullying is defined as a kind of aggressive behavior characterized by intentional harm or causing discomfort and is also accompanied by imbalanced power between the two parties (23). Bullying can manifest in different ways, such as direct assaulting, calling names, or indirect isolation from a group (24). A large cross-sectional investigation conducted in over forty countries illustrated that nearly 11% of teenager students bullied others, 13% were bullied, and 3.6% were in both roles (25). In 2019, a Chinese school-based survey of 2,579 students (aged from 10 to 17) revealed that 12.4% of them experienced bullying, of which the most common types were verbal and cyber bullying (26). Bebbington et al. reported that in people with “definite or probable” psychotic disorders, there was a clear plenitude of victimization experiences, many of which occurred in childhood (27). Furthermore, Campbell et al. found that being bullied at middle school was prominently associated with hallucinations, dissociation, and paranoia (28), and Lataster et al. reported similar findings that, after controlling for age and sex, “being bullied” was strongly related to a 3 times higher risk for having Non-clinical psychotic experiences (29).

As mentioned above, both bullying and childhood abuse and (or) neglect are traumatic experiences that contribute to the development of psychotic experiences. However, data from the Avon Longitudinal Study of Parents and Children in the UK (ALSPAC) and the Great Smoky Mountains Study in the USA (GSMS) suggested that bullying had a unique effect on mental health. Children who were only bullied by peers were more likely than children who were only maltreated (abused or neglected in family) to have depression, anxiety, and suicidal behaviors in both cohorts (30). However, it remains unclear whether bullying independently predicts PLEs, when other traumatic experiences such as abuse and (or) neglect, as well as current depression and anxiety, are controlled.

Bullying events are usually assessed by questionnaires or scales that primarily focus on the present situations (29, 31). Only two retrospective measurement tools that specifically look at past bullying events (32, 33). The Bullying Scale for Adult (BSA) is a brief self-rating questionnaire derived from the traditional bullying scale. The variety of types of bullying experiences covered in BSA allow us to investigate the dose-response effect of bullying measured by the number of endorsed items about being bullied. So far, no retrospective bullying scales for adults are available in China. Therefore, the present study aimed (1) to validate the Chinese version of Bullying Scale for Adults (C-BSA) and to retrospectively investigate the prevalence rate of bullying in Chinese university students, and (2) to determine if bullying has a unique role contributing to PLEs when other childhood trauma (abuse and neglect in family), as well as other psychopathology (depression and anxiety), are all controlled.

## Methods

### Study Participants and Procedures

Participants in the construction sample and the validation sample were all recruited in several elective courses about psychological health at a comprehensive university in North China. After teachers introduced the whole program, the voluntary students scanned a Quick Response (QR) code by WeChat (The most popular messaging app in China) and then completed a series of questionnaires. To ensure the responses validity, we embedded two quality control items (1. Please choose “not a bit”; 2. My real name is Trump) in the online questionnaires to filter out the irresponsible answers. Data with more than 5 “don't know” answers on C-BSA were also excluded.

This study was part of the project “Detection of subclinical psychotic population with traumatic symptoms and EMDR intervention”, which had been approved by the Ethics Committee of Beijing Anding Hospital, Capital Medical University. As personal identity data were not collected in this part of the project, informed consent for the current study was waived.

The English version of BSA was independently translated into Chinese by two authors (JZ & DYC). The translated scripts were reviewed and revised by a senior consultant psychiatrist (FCZ) and an associate professor in psychology (XHL) by taking into consideration of the semantic, idiomatic, and cultural equivalence of each item. All the four authors are native in Chinese and proficient in English. Then, this final version was back translated into English by a bilingual health professional (YL). The back-translated version was sent to the original author of BSA (Theresa Katharina Haidl) to ensure the equivalence. The C-BSA was administered in the construction sample to determine the items to retain, so as to generate the definitive the questionnaire (Study 1). Then the definitive questionnaire was tested in the validation sample (Study 2). Besides C-BSA, a battery of questionnaires covering traumatic experiences and emotional distress were also administered.

## Measurements

### C-BSA

The BSA was developed to evaluate adults' past bullying experiences, which was a modified version from the Bully Survey (33–35). The entire scale is divided into three parts: part A is about subjective experiences of being bullied, consisting of 13 items representing 13 types of bullying behaviors. For each item, a five-point Likert rating is used (0 = never to 4 = always) with an additional option of “don't know”. If a score other than “0” is endorsed, more detailed items about the perpetrator, time, and duration need to be answered. Part B is the personal consequences of bullying, including six items measured by a five-point Likert scale (“0—Never a problem” to “4—Always a problem”). Part C includes two items inquiring about the experience of acting as a bully perpetrator. As mentioned above, the English version of BSA was translated into Chinese to generate C-BSA.

### The 15-Item Positive Subscale of the Community Assessment of Psychic Experiences (CAPE-P15)

In order to create a shorter scale with good internal consistency for self-rating psychosis-like experience, Capra and colleagues developed CAPE-p15 (36). In 2020, Meng Sun and colleagues validated the psychometric properties of the Chinese version of CAPE-p15 in college students in China (37). The frequency and distress level related to PLEs are measured in each item. Options from 1 (never) to 4 (nearly always) are used to assess the frequency while options from 1 (not distressed) to 4 (very distressed) are for measuring distress level. Three factors were confirmed from previous studies: persecutory ideation (PI), bizarre experiences (BEs), and perceptual abnormalities (PAs) (37, 38). The Chinese version of CAPE-p15 exhibited good validity and satisfactory internal consistency (Cronbach's alpha = 0.88).

### The Chinese Version of the Childhood Trauma Questionnaire (CTQ)

The CTQ is a 28-item self-reported questionnaire developed by Bernstein for assessing childhood trauma that occurred before age 16. It consists of five subscales: emotional abuse (EA), physical abuse (PA), sexual abuse (SA), emotional neglect (EN) and physical neglect (PN) (39). Responses on a five-point Likert scale are rated from 0-never to 4-very often. The Chinese version of the CTQ showed good psychometric properties (40, 41).

### Other Scales

The Chinese version of self-rating depression scale (SDS) (42) and self-rating anxiety scale (SAS) (43) were also administered to evaluate depressive and anxiety symptoms in the past week. Both scales are rated based on a 4-point Likert scale and both have presented with good psychometric profiles in college students (44–46).

## Statistics

Data were analyzed by using SPSS version 20.0 (SPSS Inc., Chicago, IL, USA) and Mplus version 8.3. Continuous variables such as age, years of schooling, scores of CTQ, C-BSA, CAPE_FT, CAPE_PT, SDS, and SAS were summarized as Mean ± SD, whereas categorical variables (sex) were summarized as N (%). The comparisons between the construction sample and the validation sample in terms of sociodemographic, traumatic experiences, and emotional distress were carried out by using the Chi-squared test (categorical variables), *t*-test (normally distributed continuous variables) or Non-parametric test (skewed continuous variables).

Cronbach's α was calculated to determine the internal consistency. A Cronbach's α higher than 0.7 is considered acceptable, while higher than 0.9 indicates excellent (47). Exploratory factor analysis (EFA) was conducted in the construction sample to determine the reliable factor structure and which items to delete. Principal axis factoring was used to extract factors with eigenvalues >1. Promax rotation with kappa = 4 was performed (33, 48, 49). Kaiser-Meyer-Olkin (KMO) and Bartlett's test was carried out to evaluate the adequacy of factor analysis. KMO higher than 0.9 indicated perfect for factor analysis, while KMO higher 0.7 suggested acceptable (50). Items with a single factor loading no <0.4 were considered sufficient. Parallel analysis has been considered a valid method for determining the number of factors to retain (51, 52). By comparing the eigenvalues generated from the actual data with the those from a Monte-Carlo simulated matrix of the same size, the ideal number of final factors is determined. In this study, both the parallel analysis and the Kaiser's eigenvalue-greater-than-one rule were carried out.

Data of the validation sample was used for confirmatory factor analysis (CFA) to test the construct validity of C-BSA. Weighted Least Squares with Means and Variance Adjusted estimation (WLSMV) method was used as data were category variables. Fit indices including root mean square error of approximation (RMSEA), comparative fit index (CFI), Tucker-Lewis index (TLI), standardized root mean square residual (SRMR) were utilized to evaluate the model fit. RMSEA <0.08, CFI > 0.90, TLI > 0.90, SRMR <0.06 and a normed chi-square (x 2/ df) <5 were acceptable (50, 51, 53).

Spearman's correlation analysis was applied to check for associations between the scores of C-BSA and that of CTQ, CAPE-p15, SAS and SDS to assess the concurrent validity or correlations. According to the study of Sun et al. (37), the frequency score of 1.57 was the best to detect the “genuine PLEs” of the last month (54). We divided the entire sample into two groups: PLEs (frequency score ≤ 1.57 on CAPE-p15) and non-PLEs (frequency score <1.57 on CAPE-p15). To explore the independent role of bullying in predicting PLEs status (0 = no PLEs, 1 = PLEs) and to explore the dose-response effect of multiple bullying types, two logistic regression analyses with the forward LR method were conducted. In model 1, the total score of C-BSA, CTQ, SAS and SDS were entered as independent variables; in model 2, multiple experiences of being bullied (dummy coded as: 0 = no bullying, 1 = number of bullying types < = 3, 2 = number of bullying types > 3), CTQ, SAS, and SDS and sociodemographic variables were entered as independent variables.

A *P* value <0.05 was set to be significant level, and all tests were two tailed.

## Results

### Psychometric Properties of C-BSA

#### Study 1

Six hundred and forty-nine students agreed to participate in Study 1. After excluding Non-conscientious data, 629 students formed the construction sample (Table 1).

**Table 1:** Demographic and clinical characteristics of the construction sample and the validation sample.

**Variables**	**The construction sample (*N* = 629)**	**The validation sample (*N* = 629)**	**Comparisons**	
	***N* (%)/M ± SD**	***N* (%)/M ± SD**	**x^**2**^/Z/t**	***P* value**
Age	20.26 ± 3.01	20.15 ± 2.85	−0.625	0.532
Sex				
Male	256 (40.7)	229 (36.4)	2.446	0.118
Female	373 (59.3)	400 (63.6)	
Years of schooling	13.26 ± 1.24	13.25 ± 1.07	−0.290	0.772
CTQ	35.36 ± 9.97	34.87 ± 9.00	−0.031	0.975
C-BSA	5.89 ± 8.49	5.70 ± 8.46	−0.396	0.692
CAPE_FT	19.88 ± 4.92	19.95 ± 4.47	0.270	0.787
Bizarre experiences (BE)	8.96 ± 2.58	8.98 ± 2.45	0.112	0.911
Persecutory ideation (PI)	7.56 ± 2.24	7.62 ± 2.12	0.542	0.588
Perceptual abnormalities (PA)	3.36 ± 0.96	3.35 ± 0.81	–0.222	0.824
CAPE_PT	7.32 ± 7.45	7.32 ± 6.93	−0.02	0.984
SDS	43.83 ± 11.65	42.84 ± 10.95	−1.327	0.185
SAS	37.80 ± 8.97	37.84 ± 9.11	0.062	0.950

*CTQ, Childhood Trauma Questionnaire; C-BSA, the Chinese version of the Bullying Scale for Adults; CAPE_FT, Frequency Scale of the Community Assessment of Psychic Experiences; CAPE_PT, Distress Scale of the Community Assessment of Psychic Experiences; SDS, Self-rating Depression Scale; SAS, Self-rating Anxiety Scale*.

A Cronbach's α of 0.921 indicated excellent consistency (Table 2). One item had an item-scale correlation below 0.3 (0.211 for item 10) (Supplementary Table 1). One hundred and twenty-three students completed the 1-week retest and the test-retest reliability coefficient was 0.824 (*P* < 0.01). These results indicated that the C-BSA was of high reliability.

**Table 2:** Factor loadings of the Chinese version of Bullying Scale for Adults (C-BSA) (*N* = 629).

		**Factor loading (4-factor model)**					**Factor loading (3-factor model)**		
		**1**	**2**	**3**	**4**		**1**	**2**	**3**
		**Emotional**	**Interpersonal**	**Physical**	**Sexual**		**Emotional**	**Interpersonal**	**Physical and sexual**
		**abuse**	**difficulties**	**abuse**	**harassment**		**abuse**	**difficulties**	**assault**
13 Said mean things behind my back	0.695	0.959				0.604	0.867		
12 Wrote bad things about me	0.631	0.897				0.514	0.771		
5 Won't let me be a part of their group	0.530	0.581				0.526	0.711		
4 Played jokes on me	0.542	0.569				0.546	0.686		
2 Make fun of me	0.667	0.555				0.659	0.714		
6 Broke my things	0.471	0.516				0.473	0.647		
11 Won't talk to me	0.531	0.507				0.527	0.627		
1 Call me names	0.596	0.431				0.595	0.567		
17 Made difficult to study at school	0.671		0.865			0.656		0.849	
16 Made me feel bad or sad	0.714		0.668			0.714		0.662	
14 Made me feel sick	0.573		0.620			0.571		0.618	
18 Made me not go to school	0.369		0.495			0.392		0.510	
15 I couldn't make friends	0.515		0.476			0.518		0.485	
19 I had problems with my family	0.365		0.446			0.342		0.462	
8 Assaulted me (except sexually)/robbed me	0.450			0.728		0.344			0.505
3 Said they will do bad thing to me	0.582			0.622		0.546			0.482
7 Attacked me physically (except sexually)	0.446			0.532		0.425			0.409
9 Sexually harassed me	0.776				0.820	0.431			0.640
10 Sexually assaulted me	0.452				0.592	0.386			0.689
% Variance Explained		40.547	6.882	4.836	3.388		40.382	6.293	4.741
		Total = 55.653					Total = 51.415	
Cronbach's alpha		0.899	0.833	0.715	0.745		0.899	0.833	0.751
		Total = 0.921					Total = 0.921	

EFA was performed in the construction sample. A Kaiser–Meyer–Olkin (KMO) of 0.907 and the Bartlett's test of sphericity (*P* < 0.001) supported the adequacy of factor analysis.

(1) Four factors with eigenvalues of more than 1 were extracted which explained the 55.65% of the cumulative variance (four-factor model). Factor names, items, communalities, and loadings were showed in Table 2. Factor 1 “Emotional abuse” composed of items 1, 2, 4, 5, 6, 11, 12, 13 which explained 40.55% of the variance; factor 2 “Interpersonal difficulties” composed of items 14 to 19 which accounted for 6.88% of the variance; factor 3 “Physical abuse” including item 3, 7, 8 which explained 4.84% of the variance; and factor 4 “Sexual harassment” including item 9 and 10 which accounted for 3.39% of the variance (Table 2).

(2) In parallel analysis, only eigenvalues higher than the upper limit of 95% confidence intervals (CIs) of the simulated datasets were retained, resulting in a three-factor model. The three factors' eigenvalues were 8.130, 1.737, and 1.363, respectively. The latent variables explained 40.382, 6.293, and 4.741 percent of the variance, accounting for 51.415 percent of the total variance. The factor distribution differed slightly from that of the four-factor model, with two same factors and the third factor being the combination of factors 3 and 4 of the four-factor model (Table 2).

#### Study 2

Six hundred and sixty-seven students agreed to participate in Study 2. After excluding Non-conscientious data, 629 students formed the validation sample (Table 1). In comparison of the demographic and clinical characteristics between the two samples, no significant differences were found.

Confirmatory Factorial Analysis (CFA) in the validation sample was carried out to verify the four-factor model and the three-factor model revealed in EFA. The four-factor model showed good fit for the data. The fit indices of the model were χ^2^ = 406.637, *p* < 0.001, RMSEA = 0.053, SRMR = 0.049, CFI = 0.980, and TLI = 0.977. The model was considered good, since CFI and TLI exceeded 0.95, and RMSEA was <0.08. These results suggested that the four-factor model gives a close fit to our sample data. As for the three-factor model, all the model fits seemed adequate (RMSEA = 0.057, 95% CI = 0.051 / 0.063; TLI = 0.974, CFI = 0.978, χ2/df = 3.009, SRMR = 0.06).

### Bullying and Psychotic-Like Experiences in the Whole Sample

In combining the construction and the validation samples (the whole sample), 699 (55.56%) reported bully related experiences, including 504 (40.06%) as victims only, 25 (1.99%) as perpetrator only, and 170 (13.51%) as both victims and perpetrators. Regarding experiences of being bullied, the most endorsed item was item 4 “Played jokes on me”, while the least endorsed item was item 10 “Sexually assaulted me”. The frequency distribution of endorsed items about being bullied are shown in Supplementary Table 2.

There was a significantly higher proportion of individuals as bully victims (50.7 vs. 37.8%) and as both victim and perpetrator (28.8 vs. 10.3%) in PLEs than that in non-PLEs (Supplementary Table 3). Data from the two samples showed a significant correlation between the total scores of C-BSA and CTQ, CAPE-p15, SDS, and SAS (Supplementary Table 4). The correlation coefficient between BSA and CTQ was 0.31 indicating moderate correlation.

Regarding the multivariate logistic analyses, both model 1 and model 2 revealed that bullying significantly and independently predicted PLEs status. In model 1, the total score of C-BSA (OR = 1.070, *P* < 0.001, 95% CI: 1.049–1.092), CTQ (OR = 1.022, *P* = 0.023, 95% CI: 1.003–1.041), and SAS (OR = 1.091, *P* < 0.001, 95% CI: 1.067–1.115) were significant predictors of PLEs. In model 2, PLEs were significantly predicted by multiple experiences of being bullied (dummy #1: OR = 2.012, *P* = 0.003, 95% CI: 1.271–3.184; dummy #2: OR = 3.712, *P* < 0.001, 95% CI: 2.472–5.573), the total score of CTQ (OR = 1.032, *P* < 0.001, 95% CI: 1.014–1.050) and SAS (OR = 1.097, *P* < 0.001, 95% CI: 1.074–1.121) (Table 3).

**Table 3:** Multivariate logistic regression analyses for predicting psychosis-like experiences (PLEs) (*N* = 1,258).

**Variable**	**Dummy variable**	**B**	**S.E**.	**Wald**	**df**	**Sig**.	**Exp (B)**	**95% C.I. for EXP(B)**	
								**Lower**	**Upper**
**Model 1**									
Age		−0.150	0.038	15.227	1.000	0.000	0.861	0.798	0.928
CTQ		0.022	0.009	5.177	1.000	0.023	1.022	1.003	1.041
C-BSA		0.068	0.010	44.380	1.000	0.000	1.070	1.049	1.092
SAS		0.087	0.011	60.746	1.000	0.000	1.091	1.067	1.115
Constant		−3.346	0.760	19.401	1.000	0.000	0.035		
**Model 2**									
Age		−0.131	0.036	12.937	1.000	0.000	0.878	0.817	0.942
Multiple experience of being bullied	0 (reference)			40.201	2.000	0.000			
	1–3 types (dummy #1)	0.699	0.234	8.901	1.000	0.003	2.012	1.271	3.184
	4–13 types (dummy #2)	1.311	0.207	40.012	1.000	0.000	3.712	2.472	5.573
SAS		0.093	0.011	72.965	1.000	0.000	1.097	1.074	1.121
CTQ		0.032	0.009	12.433	1.000	0.000	1.032	1.014	1.050
Constant		−4.536	0.721	39.619	1.000	0.000	0.011		

*CTQ, Childhood Trauma Questionnaire; C-BSA, the Chinese version of the Bullying Scale for Adults; SAS, Self-rating Anxiety Scale*.

**Figure 1:**
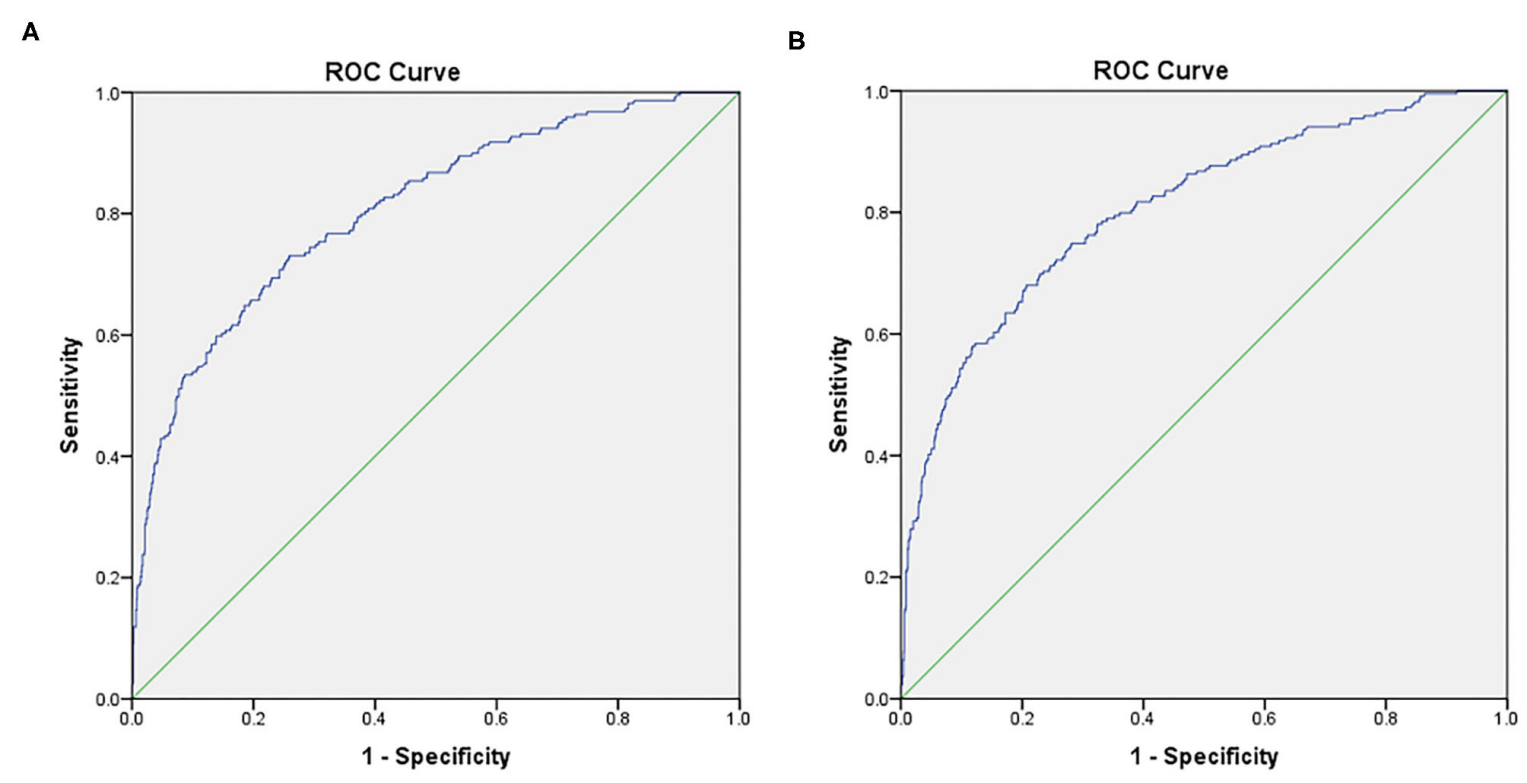
Receiver operating characteristic (ROC) curves for the two models of multivariate logistic regression analyses in predicting the presence of PLEs. The area under the curve (AUC) was estimated to be 0.808 (95% CI: 0.775 to 0.841) for the first model with a sensitivity of 73.1% and a specificity of 74.1% **(A)**; and was 0.807 (95% CI: 0.774 to 0.840) for the second model with a sensitivity of 68.0% and a specificity of 79.3% **(B)**.

Figure 1 shows the ROC curves for the predicted probabilities from the two final models. The area under the curve (AUC) was estimated to be 0.808 (95% CI: 0.775 to 0.841) for the first model with a sensitivity of 73.1% and a specificity of 74.1% (Figure 1A); and was 0.807 (95% CI: 0.774 to 0.840) for the second model with a sensitivity of 68.0% and a specificity of 79.3% (Figure 1B).

## Discussion

To the best of our knowledge, this is the first study that has ever investigated whether past bullying experiences independently contribute to the presence of PLEs. In this study, more than half of the participant students reported at least one past bullying experience based on the endorsed items on C-BSA. Our findings have confirmed the hypothesis that bullying has a unique contribution to the presence of PLEs, which was associated with 2.0 or 3.7 times the risk for the presence of PLEs (numbers of bullying types < = 3 or > 3, respectively) even after the total scores of CTQ, SDS, and SAS were all controlled.

The overall prevalence rate of bullying related behaviors in our sample is 55.56%. Specifically, 1.99% students bullied others, 13.51% reported as both a victim and a perpetrator, and 40.06% reported being bullied. Similar findings can be found in another report which investigated 95,873 students from 85 vocational schools in Southeast China (55). However, the self-reported prevalence of bullying victimization varied dramatically across different studies, ranging from 2 to 66% in Mainland China, possibly due to discrepancies in sampling methods, measurements of bullying, and study designs (56).

Overall, the C-BSA showed good preliminary validity and reliability in the present sample. Regarding internal consistency, item 10 “Sexually assaulted me” had an item-scale correlation below 0.3. This may be explained by the fact that this was the least endorsed item about being bullied. Only 34 (2.70%) students endorsed item 10. The result was interesting when compared with Haidl et al. (33)'s stud. In their study, item 10 showed an item scale correlation of <0.3 in Italy and Finland. This item has not been removed from the C-BSA in the current study because every student who endorsed this item had a frequency score ≤ 1.57 on CAPE-p15, suggesting clinical relevance of the item with PLEs. However, these findings need to be re-examined in larger samples in the future.

The correlation coefficient between BSA and CTQ was 0.31 indicating moderate correlation. This correlation coefficient is well below that in Haidl et al. (33)'s study, in which the coefficient ranged from 0.458 in Finnish sample to 0.680 in German sample. This discrepancy could be due to the difference in sample composition in terms of their clinical settings. In the present study, we only recruited Non-clinical college students while the sample of Haidl et al.'s study consisted of a majority of clinical patients. Moreover, these findings also suggest that bullying may have a unique role in the context of traumatic experiences in Chinese culture, which is relatively independent of family abuse and (or) neglect (indicated by CTQ).

Several lines of evidence have shown that both maltreatment in family and bully victimization in schools would increase the expression of psychosis phenotype (57–59). Individuals at ultra-high risk for psychosis often reported various types of childhood trauma and bullying experiences, as well as anxiety and depression (60–62). In line with these reports, score of CAPE-p15 was also positively correlated with that of SDS, SAS, C-BSA, and CTQ in the present study. Exposure to childhood adversities could disrupt the function of hypothalamic-pituitary-adrenal (HPA) and activate dopaminergic circuits, leading to a vulnerability to psychosis (63). Within the framework of stress-vulnerability model, various trauma are all transdiagnostic risk factors, leading to subsequent mental disorders through common biological pathways (63). In this regard, the risk of psychosis would increase with the accumulation of early life adversities (64), regardless of the type of trauma.

However, the multivariate logistic regression revealed that bullying was associated with 2.0 or 3.7 times the of risk for the presence of PLEs (numbers of bullying types < = 3 or > 3, respectively) even after controlling for CTQ, SDS, and SAS scores, suggesting the contribution of bullying to PLEs is independent of the effect of maltreatment in the family (measured by CTQ). Children being bullied usually have an excessive appraisal of the victimization (65), and an increase of the psychotic-like or paranoid reactivity to stress (66, 67). Beyond the field of psychosis, the unique role of bullying has already been implicated in two cohorts studies (ALSPAC and GSMS), in which children bullied by peers were more likely to have depression, anxiety, and suicidal behaviors than children who were only maltreated in family (30). The dose-response relationship between the numbers of bullying types and the presence of PLEs was a new finding, although the association between the severity of bullying and PLEs has already been established (31). It has also been demonstrated that the PLEs would deteriorate (68) and the transition to psychosis are likely to happen with the increase of bullying severity (31).

Multivariate logistic regression revealed that maltreatment in family (indicated by scores of CTQ) and anxiety (indicated by scores of SAS) also independently correlated with PLEs, which was consistent with previous reports (69–71). However, depression was no longer an independent risk factor for PLEs when traumatic experiences and anxiety were all controlled. Fisher et al. (72) discovered in a student sample that anxiety played a mediating role between emotional abuse and paranoia. Yamasaki et al. (73) demonstrated that depression did not exhibit significant mediation effects between peer victimization and hallucinations. However, inconsistent findings were also reported in previous literatures (74, 75). Further studies are warranted to fully unravel the association of depression and PLEs.

The results should be interpreted with caution due to potential methodological limitations. First, we did not conduct a structured interview for the college students to verify their PLEs in the present study. Subjective reports of PLEs have been linked to a higher false positive rate in the community population (76, 77). Second, all the participants were Non-help seeking college students, which limits the generalizability of the findings to clinical populations. Third, although C-BSA reflects past bullying experiences, the cross-sectional design could not explore dynamic interaction between PLEs, various traumatic experiences, and other psychopathology.

In conclusion, the key findings of this study include (1) C-BSA has showed good psychometric properties in university students in China; (2) The contribution of past bullying experiences to the presence of PLEs seems to be independent of other childhood trauma, current depression, and anxiety; and (3) There is a dose-response relationship between the numbers of bullying types and the presence of PLEs.

The study shed light on the differential contributions between bullying and maltreatment in family to the increased risk of psychosis. By addressing the unique effects of past bullying experiences, the findings of the study would lend support to the future development of individualized therapeutic strategies for individuals with PLEs.

## Data Availability Statement

The data that support the findings of this study are available from the corresponding author upon reasonable request.

## Ethics Statement

The studies involving human participants were reviewed and approved by the Ethics Committee of Beijing Anding Hospital, Capital Medical University. Written informed consent from the participants' legal guardian/next of kin was not required to participate in this study in accordance with the national legislation and the institutional requirements.

## Author Contributions

JZ: conceptualization, methodology, formal analysis, and writing—original draft. X-HLu: methodology, formal analysis, and data curation. YL: resources and writing—review and editing. NW: formal analysis and writing—review and editing. D-YC: investigation, software, and data curation. I-AL: investigation, software, and validation. X-HLi: supervision, methodology, and writing—review and editing. F-CZ: conceptualization, project administration, methodology, formal analysis, and writing—review and editing. C-YW: conceptualization, supervision, funding acquisition, and writing—review and editing. All authors contributed to the article and approved the submitted version.

## Funding

This work was funded by the National Science and Technology Major Project for IND (investigational new drug) (Grant No. 2018ZX09201-014) and the Beijing Municipal Administration of Hospitals Clinical Medicine Development of special funding (ZYLX201815).

## Conflict of Interest

The authors declare that the research was conducted in the absence of any commercial or financial relationships that could be construed as a potential conflict of interest.

## Publisher's Note

All claims expressed in this article are solely those of the authors and do not necessarily represent those of their affiliated organizations, or those of the publisher, the editors and the reviewers. Any product that may be evaluated in this article, or claim that may be made by its manufacturer, is not guaranteed or endorsed by the publisher.
