# Supplementary material for: The Unique Contribution of Past Bullying Experiences to the Presence of Psychosis-Like Experiences in University Students
Source: Front Psychiatry. 2022 Apr 28;13:839630. doi: 10.3389/fpsyt.2022.839630 (PMC9096161; doi:10.3389/fpsyt.2022.839630)
Supplement: Supplementary file 1 [file Data_Sheet_1.doc]

**Supplementary table 1. The item-scale and item-dimension correlations (N=629).**

| Dimensions | Items | Item correlation with the total score | Item correlation with the total dimension score |
| --- | --- | --- | --- |
| Emotional abuse | 1 | 0.649 | 0.691 |
|  | 2 | 0.752 | 0.805 |
|  | 4 | 0.785 | 0.847 |
|  | 5 | 0.586 | 0.623 |
|  | 6 | 0.555 | 0.591 |
|  | 11 | 0.658 | 0.690 |
|  | 12 | 0.558 | 0.601 |
|  | 13 | 0.643 | 0.697 |
| Interpersonal difficulties | 14 | 0.658 | 0.719 |
|  | 15 | 0.661 | 0.738 |
|  | 16 | 0.823 | 0.912 |
|  | 17 | 0.624 | 0.720 |
|  | 18 | 0.337 | 0.382 |
|  | 19 | 0.450 | 0.517 |
| Physical abuse | 3 | 0.541 | 0.825 |
|  | 7 | 0.490 | 0.780 |
|  | 8 | 0.303 | 0.480 |
| Sexual harassment | 9 | 0.325 | 0.960 |
|  | 10 | 0.211 | 0.696 |

**Supplementary table 2. The frequency distribution of endorsed items about being bullied (n (%)).**

| **Others…** | **Never** | **Rarely** | **Sometimes** | **Often** | **Always** | **Don’t know** |
| --- | --- | --- | --- | --- | --- | --- |
| Call me names | 960(76.31) | 186(14.79) | 71(5.64) | 28(2.23) | 7(0.56) | 6(0.48) |
| Make fun of me | 869(69.08) | 244(19.40) | 101(8.03) | 33(2.62) | 10(0.79) | 1(0.08) |
| Said they will do bad thing to me | 1068(84.90) | 147(11.69) | 32(2.54) | 7(0.56) | 2(0.16) | 2(0.16) |
| Played jokes on me | 773(61.45) | 247(19.63) | 156(12.40) | 63(5.01) | 16(1.27) | 3(0.24) |
| Won't let me be a part of their group | 995(79.09) | 157(12.48) | 69(5.48) | 18(1.43) | 15(1.19) | 4(0.32) |
| Broke my things | 1045(83.07) | 147(11.69) | 53(4.21) | 10(0.79) | 2(0.16) | 1(0.08) |
| Attacked me physically (except sexually) | 1087(86.41) | 125(9.94) | 38(3.02) | 5(0.40) | 3(0.24) | 0(0.00) |
| Assaulted me (except sexually) / robbed me | 1206(95.87) | 34(2.70) | 11(0.87) | 6(0.48) | 1(0.08) | 0(0.00) |
| Sexually harassed me | 1185(94.20) | 51(4.05) | 19(1.51) | 3(0.24) | 0(0.00) | 0(0.00) |
| Sexually assaulted me | 1224(97.30) | 21(1.67) | 10(0.79) | 3(0.24) | 0(0.00) | 0(0.00) |
| Won’t talk to me | 963(76.55) | 170(13.51) | 87(6.92) | 24(1.91) | 11(0.87) | 3(0.24) |
| Wrote bad things about me | 1030(81.88) | 140(11.13) | 50(3.97) | 18(1.43) | 3(0.24) | 17(1.35) |
| Said mean things behind my back | 879(69.87) | 218(17.33) | 102(8.11) | 29(2.31) | 7(0.56) | 23(1.83) |

**Supplementary table 3. Comparison of demographic characteristics and bullying behaviors between PLEs and Non-PLEs.**

| Variables | Total (N=1258) | PLEs (N=219) | Non-PLEs (N=1039) | Comparisons | |
| --- | --- | --- | --- | --- | --- |
|  | N(%)/M± SD | N(%)/M± SD | N(%)/M± SD | x^2^/Z | P-value |
| Age | 20.20 ± 2.93 | 20.27 ± 2.58 | 20.19 ± 3.00 | -0.508 | 0.611 |
| Sex  Male   Female | 485 (38.6) 773 (61.4) | 89 (40.6) 130 (59.4) | 396 (38.1) 643 (61.9) | 0.487 | 0.485 |
| Years of schooling | 13.26 ± 1.16 | 13.33 ± 1.23 | 13.24 ± 1.14 | -0.510 | 0.610 |
| CTQ | 35.11 ± 9.49 | 41.40 ± 12.67 | 33.79 ± 8.08 | -9.248 | 0.000 |
| C-BSA | 5.79 ± 8.47 | 13.16 ± 12.06 | 4.24 ± 6.52 | -12.259 | 0.000 |
| Four bullying types  No-Status  Victim  Bully-Victim  Bully | 559 (44.4) 504 (40.1) 170 (13.5) 25 (2.0) | 41 (18.7) 111 (50.7) 63 (28.8) 4 (1.8) | 518 (49.9) 393 (37.8) 107 (10.3) 21 (2.0) | 92.613 | 0.000 |
| CAPE_FT | 19.91 ± 4.70 | 27.74 ± 4.90 | 18.26 ± 2.46 | -23.391 | 0.000 |
| CAPE_PT | 7.32 ± 7.19 | 18.83 ± 7.46 | 4.89 ± 4.14 | -22.002 | 0.000 |
| SDS | 43.34 ± 11.31 | 51.94 ± 11.92 | 41.52 ± 10.31 | -11.476 | 0.000 |
| SAS | 37.82 ± 9.03 | 45.75 ± 11.04 | 36.15 ± 7.56 | -12.406 | 0.000 |

Abbreviations: CTQ, Childhood Trauma Questionnaire; C-BSA, the Chinese version of the Bullying Scale for Adults; CAPE_FT, Frequency Scale of the Community Assessment of Psychic Experiences; CAPE_PT, Distress Scale of the Community Assessment of Psychic Experiences; SDS, Self-rating Depression Scale; SAS, Self-rating Anxiety Scale.

| **Supplementary table 4. Bivariate correlation matrix of the clinical characteristics (N = 1258).**   |  | C-BSA | CTQ | CAPE_FT | CAPE_PT | SDS | SAS | | --- | --- | --- | --- | --- | --- | --- | | C-BSA | 1 |  |  |  |  |  | | CTQ | .330^**^ | 1 |  |  |  |  | | CAPE_FT | .490^**^ | .275^**^ | 1 |  |  |  | | CAPE_PT | .518^**^ | .283^**^ | .964^**^ | 1 |  |  | | SDS | .330^**^ | .566^**^ | .350^**^ | .364^**^ | 1 |  | | SAS | .362^**^ | .521^**^ | .381^**^ | .392^**^ | .772^**^ | 1 | |  |
| --- | --- | --- | --- | --- | --- | --- | --- | --- | --- | --- | --- | --- | --- | --- | --- | --- | --- | --- | --- | --- | --- | --- | --- | --- | --- | --- | --- | --- | --- | --- | --- | --- | --- | --- | --- | --- | --- | --- | --- | --- | --- | --- | --- | --- | --- | --- | --- | --- | --- | --- |

Note: CTQ, Childhood Trauma Questionnaire; C-BSA, the Chinese version of the Bullying Scale for Adults; CAPE_FT, Frequency Scale of the Community Assessment of Psychic Experiences; CAPE_PT, Distress Scale of the Community Assessment of Psychic Experiences; SDS, Self-rating Depression Scale; SAS, Self-rating Anxiety Scale.

**Supplementary table 5. Percentage distributions for the additional answer options in the C-BSA (n(%)).**

| **Others…** | **Other students/ colleagues** | **School staffs / coach / instructor/ supervisor** | **Family member/partner** | **Neighbors (living place)** |
| --- | --- | --- | --- | --- |
| Call me names | 254(85.23) | 52(17.45) | 72(24.16) | 14(4.70) |
| Make fun of me | 357(91.77) | 52(13.37) | 52(13.37) | 17(4.37) |
| Said they will do bad thing to me | 172(90.53) | 15(7.89) | 17(8.95) | 10(5.26) |
| Played jokes on me | 466(96.08) | 35(7.22) | 49(10.10) | 24(4.95) |
| Won't let me be a part of their group | 246(93.54) | 24(9.13) | 5(1.90) | 11(4.18) |
| Broke my things | 198(92.96) | 13(6.10) | 26(12.21) | 7(3.29) |
| Attacked me physically (except sexually) | 149(87.13) | 14(8.19) | 36(21.05) | 5(2.92) |
| Assaulted me (except sexually) / robbed me | 43(82.69) | 2(3.85) | 8(15.38) | 5(9.62) |
| Sexually harassed me | 42(57.53) | 11(15.07) | 12(16.44) | 18(24.66) |
| Sexually assaulted me | 15(44.12) | 7(20.59) | 7(20.59) | 12(35.29) |
| Won’t talk to me | 280(94.92) | 22(7.46) | 34(11.53) | 15(5.08) |
| Wrote bad things about me | 222(97.37) | 12(5.26) | 11(4.82) | 6(2.63) |
| Said mean things behind my back | 361(95.25) | 22(5.80) | 23(6.07) | 21(5.54) |
|  |  |  |  |  |
|  | **At least for 1 month** | **Past 12 months** | **Before age 17** |  |
| Call me names | 32(10.74) | 41(13.76) | 251(84.23) |  |
| Make fun of me | 39(10.03) | 47(12.08) | 330(84.83) |  |
| Said they will do bad thing to me | 14(7.37) | 19(10.00) | 165(86.84) |  |
| Played jokes on me | 68(14.02) | 95(19.59) | 370(76.29) |  |
| Won't let me be a part of their group | 26(9.89) | 46(17.49) | 208(79.09) |  |
| Broke my things | 19(8.92) | 26(12.21) | 183(85.92) |  |
| Attacked me physically (except sexually) | 11(6.43) | 12(7.02) | 154(90.06) |  |
| Assaulted me (except sexually) / robbed me | 5(9.62) | 4(7.69) | 46(88.46) |  |
| Sexually harassed me | 9(12.33) | 9(12.33) | 59(80.82) |  |
| Sexually assaulted me | 5(14.71) | 6(17.65) | 26(76.47) |  |
| Won’t talk to me | 51(17.29) | 69(23.39) | 218(73.90) |  |
| Wrote bad things about me | 20(8.77) | 29(12.72) | 199(87.28) |  |
| Said mean things behind my back | 53(13.98) | 73(19.26) | 305(80.47) |  |

**Supplementary table 6. The impact of bullying (n (%)).**

|  | **Never a problem** | **Rarely a problem** | **Sometimes a problem** | **Often a problem** | **Always a problem** |
| --- | --- | --- | --- | --- | --- |
| Made me feel sick | 926(73.61) | 207(16.45) | 104(8.27) | 12(0.95) | 9(0.72) |
| I couldn’t make friends | 938(74.56) | 182(14.47) | 100(7.95) | 27(2.15) | 11(0.87) |
| Made me feel bad or sad | 754(59.94) | 225(17.89) | 177(14.07) | 71(5.64) | 31(2.46) |
| Made difficult to study at school | 975(77.50) | 160(12.72) | 89(7.07) | 21(1.67) | 13(1.03) |
| Made me not go to school | 1180(93.80) | 48(3.82) | 25(1.99) | 2(0.16) | 3(0.24) |
| I had problems with my family | 1103(87.68) | 80(6.36) | 45(3.58) | 16(1.27) | 14(1.11) |

**Supplementary table 7. The frequency and duration of bullying others (n (%)).**

| **Never** | **Rarely** | **Sometimes** | **Often** | **Always** | **Don’t know** |
| --- | --- | --- | --- | --- | --- |
| 1058(84.10) | 155(12.32) | 37(2.94) | 1(0.08) | 2(0.16) | 5(0.40) |
|  |  |  |  |  |  |
| **At least for a month** | **Past 12 months** | **Before age 17** |  |  |  |
| 11(5.50) | 23(11.50) | 166(83.00) |  |  |  |
